# Thermally Reduced Graphene Oxide Membranes Revealed Selective Adsorption of Gold Ions from Mixed Ionic Solutions

**DOI:** 10.3390/ijms241512239

**Published:** 2023-07-31

**Authors:** Yu Qiang, Siyan Gao, Yueyu Zhang, Shuai Wang, Liang Chen, Liuhua Mu, Haiping Fang, Jie Jiang, Xiaoling Lei

**Affiliations:** 1 School of Physics and School of Material Science and Engineering, East China University of Science and Technology, Shanghai 200237, China; y10200135@mail.ecust.edu.cn (Y.Q.); y10210069@mail.ecust.edu.cn (S.G.); y10200137@mail.ecust.edu.cn (S.W.); fanghaiping@sinap.ac.cn (H.F.); 2 Wenzhou Institute, University of Chinese Academy of Sciences, Wenzhou 325001, China; zhangyy@wiucas.ac.cn (Y.Z.); muliuhua@hotmail.com (L.M.); 3 University of Chinese Academy of Sciences, Beijing 100049, China; 4 School of Physical Science and Technology, Ningbo University, Ningbo 315211, China; liang_chen05@126.com

**Keywords:** rGO membranes, adsorption, selectivity, gold recycling, adsorptive−reduction, two-dimensional materials

## Abstract

The recovery of gold from water is an important research area. Recent reports have highlighted the ultrahigh capacity and selective extraction of gold from electronic waste using reduced graphene oxide (rGO). Here, we made a further attempt with the thermal rGO membranes and found that the thermal rGO membranes also had a similarly high adsorption efficiency (1.79 g gold per gram of rGO membranes at 1000 ppm). Furthermore, we paid special attention to the detailed selectivity between Au^3+^ and other ions by rGO membranes. The maximum adsorption capacity for Au^3+^ ions was about 16 times that of Cu^2+^ ions and 10 times that of Fe^3+^ ions in a mixture solution with equal proportions of Au^3+^/Cu^2+^ and Au^3+^/Fe^3+^. In a mixed-ion solution containing Au^3+^:Cu^2+^:Na^+^:Fe^3+^:Mg^2+^ of printed circuit board (PCB), the mass of Au^3+^:Cu^2+^:Na^+^:Fe^3+^:Mg^2+^ in rGO membranes is four orders of magnitude higher than the initial mass ratio. A theoretical analysis indicates that this selectivity may be attributed to the difference in the adsorption energy between the metal ions and the rGO membrane. The results are conducive to the usage of rGO membranes as adsorbents for Au capture from secondary metal resources in the industrial sector.

## 1. Introduction

Gold is widely used in electronic devices on connectors and contact points because of its high electrical conductivity and corrosion-resistant properties. One of the most important precious metals, statistics from 2012 show that around 300 tons of gold were used in electronic industries along with other precious and strategic metals like Ag, Pd, Pt, Nb, Ta, etc. [1]. Printed circuit boards (PCBs) contain more precious metals than the ores in mines. Especially, the level of gold in PCBs is about 35–50 times higher than that of original minerals [2]. PCBs amount to around 40 million tons per year, with a 5% increase every year [2,3]. Metal recycling from secondary metal resources is an important way to replace future primary metal resources [4]. The most widely used methods for the recovery of metals from PCBs are mechanical processing (crushing, jigging, shaping and electrostatic separation), pyrometallurgical techniques (heating and smelting) and hydrometallurgical methods (pickling) [5,6,7]. Although these techniques are relatively inexpensive and quick for gold recovery, they exhibit low efficiency and low selectivity, and there are health risks associated with these conventional methods [8]. Currently, 50% of e-waste ends up in landfills due to a lack of recycling programs with high selectivity and productivity [9,10].

It is compelling to develop sustainable and eco-friendly technologies with higher selectivity and extraction capacity for gold extraction from the leachate of waste PCBs [4,6]. Various novel gold extraction methods have been explored recently [11,12,13,14,15]. Xue et. al. reported a record-breaking extraction rate, with high Au^3+^ removal efficiency (>99%) within seconds (less than 45 s), a competitive capacity (1.6 g/g) with a highly porous metal-organic framework (MOF)–polymer composite and BUT-33–poly(para-phenylenediamine) for gold extraction from e-waste [12]. By a new method based on precisely controlling the reciprocal transformation of the second-sphere-coordinated adducts formed between β-CD and [AuBr_4_]^−^ anions, the efficiency of gold recovery reaches 99.8% when dibutyl carbitol is deployed as the additive [13]. By regulating the hydrogen-bond nanotrap within the pore of covalent organic frameworks (COFs), Qiu et al. reported a novel strategy to achieve precise recognition and separation of gold, which can efficiently capture Au^3+^ with fast kinetics, high selectivity and an uptake capacity of 1.725 g/g [14]. By molecular recognition technology, Love and co-workers developed a simple tertiary diamide that precipitates gold selectively from aqueous acidic solutions, including from aqua regia solutions of electronic waste [15]. Li et al. reported an exceptionally high gold extraction capacity of chemically reduced graphene oxide (rGO), reaching 1.85 g/g when extracting gold from its 10 ppm solution at 25 °C, combined with an ability to extract gold at minute concentrations, down to parts per trillion, and high selectivity [11]. More interestingly, the rGO exclusively extracts gold without contamination of the other 14 coexisting metallic elements generally seen in e-waste, manifesting excellent extraction selectivity. These superior performances will open a novel, green and efficient route to address the challenges of gold sustainability and global e-waste accumulation [13,16].

Inspired by the work of Li et al. [11], we further attempted to use thermally rGO membranes to the adsorption properties of Au^3+^ in solution using an rGO membrane. As we are aware, rGO membranes are highly user-friendly in practical applications. They can be easily recovered and desorbed after the soaking process. We found that 1 g of rGO membrane can extract 1.79 g of gold from 1000 ppm gold solution within 48 h. As we known, PCB contains a large number of metal ions such as Cu^2+^, Fe^3+^ and Mg^2+^, which are mixed with Au^3+^ to form a complex environment. Thus, it is necessary to comprehensively understand the selectivity of Au^3+^ by rGO membrane. We systematically studied the selective adsorption of rGO membranes to Au^3+^ in double-ion and multi-ion solutions. When the membrane was immersed in a mixture solution with equal proportions of Au^3+^/Cu^2+^ and Au^3+^/Fe^3+^, the maximum adsorption capacity for Au^3+^ ions was about 16 times that of Cu^2+^ ions and 10 times that of Fe^3+^ ions. Subsequently, we used the rGO membranes to adsorb Au^3+^ ions in the mixed-ion solution, and the results showed that the concentration of Au^3+^ after adsorption was four times higher than the initial concentration, indicating that the rGO membranes had excellent selective adsorption of Au^3+^ in secondary metal resources. The mechanism is also explained by the difference in absorption energies of the corresponding ions obtained by density functional theory (DFT) calculations.

## 2. Results and Discussion

### 2.1. The Adsorption of rGO Membranes on Au^3+^ Ions

**Figure 1:**
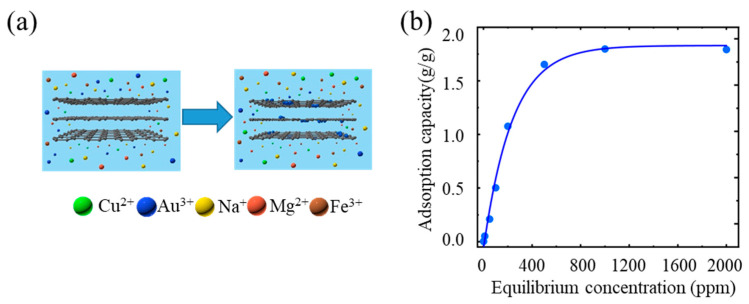
Adsorption capacity of Au^3+^ ions using rGO membranes. (**a**) Schematic diagram of the adsorption process. (**b**) Gold adsorption isotherms of rGO membranes after 48 h. The Au^3+^ adsorption capacity of rGO membranes can reach up to 1.79 g/g.

To study the Au^3+^ adsorption capacity of an rGO membrane in solution, the dry thermal reduction rGO membranes were immersed in a series of AuCl_3_ solutions from 50 ppm to 2000 ppm to obtain the rGO membrane, as shown in Figure 1a. The adsorption capacity of rGO membranes was defined as the mass ratio between the adsorbed Au^3+^ and the original rGO membrane. From the isothermal adsorbing experiment, Figure 1a shows that the maximum adsorption capacity of Au^3+^ ions is 0.0026 g/g, 0.051 g/g, 0.27 g/g, 0.55 g/g, 1.07 g/g, 1.62 g/g, 1.79 g/g and 1.79 g/g with the concentrations of 0.5 ppm, 10 ppm, 50 ppm, 100 ppm, 200 ppm, 500 ppm, 1000 ppm and 2000 ppm, respectively. The Au^3+^ adsorption capacity of rGO membranes can reach up to 1.79 g/g, which is similar to the capacity of 1.85 g/g reported by Li et al. at 25 °C [11], and which is much higher than the adsorption capacity of activated carbon at ~0.04 g/g [17], ion exchange resin at ~0.05 g/g [18], nanofibrous membranes at ~0.25 g/g [19] and porous porphyrin polymer at ~1.62 g/g [20] (Table 1). In addition to the adsorption capacity, we also paid attention to the adsorption efficiencies at different concentrations, as shown in Figure S1. At concentrations of 0.5 ppm, 10 ppm, 50 ppm and 100 ppm, the rGO membrane adsorbs almost all gold ions in the solution within 4 h, and the adsorption efficiency is 97%, 96%, 94% and 96%, respectively (Figures S1−S5). In a solution with a concentration greater than 100 ppm, the rGO membrane completed 40% of its adsorption capacity within the first 4 h.

### 2.2. Excellent Selective Adsorption of rGO Membranes to Au^3+^ in Mixed Solutions

**Figure 2:**
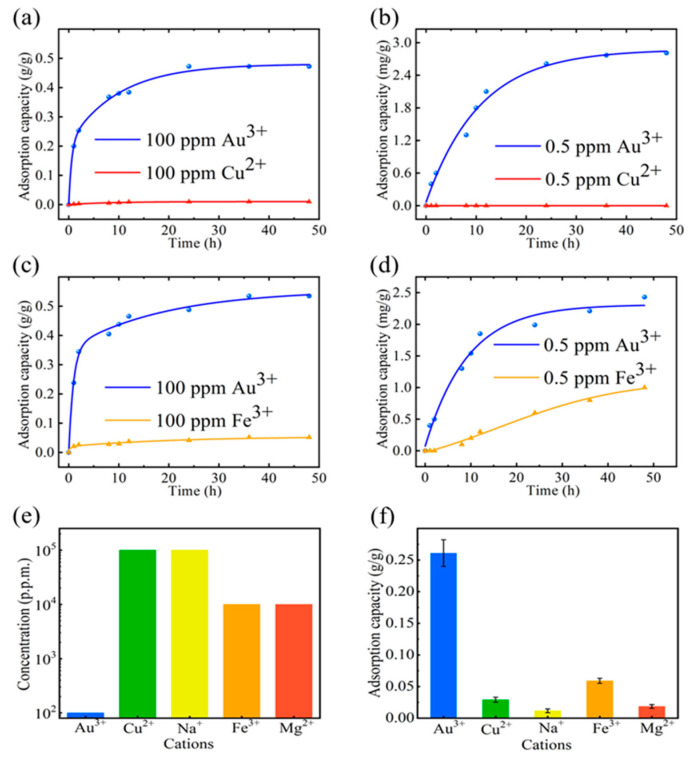
Excellent selective adsorption of rGO membranes to Au^3+^ in mixed solutions (**a**,**b**). The adsorption capacity of Au^3+^ ions in Au^3+^/Cu^2+^ mixed-ion solutions (100 ppm/0.5 ppm). (**c**,**d**) The adsorption capacity of Au^3+^ ions in Au^3+^/Fe^3+^ mixed-ion solutions (100 ppm/0.5 ppm). (**e**) The initial concentration ratio of the configured mixed-ion solution (denoted R_i_) for Au^3+^:Cu^2+^:Na^+^:Fe^3+^:Mg^2+^ as R_i_ = 1:1000:1000:100:100, where the concentration of Au^3+^ was 100 ppm. (**f**) The adsorption capacity of 5 kinds of ions in the configured mixed-ion solution by rGO membranes. The corresponding mass ratio in the rGO membranes (denoted R_f_) of Au^3+^:Cu^2+^:Na^+^:Fe^3+^:Mg^2+^ were R_f_ = 1:0.11:0.06:0.22:0.07.

As we known, PCB leachates contain as many as 60 different elements and complex components, such as Cu^2+^, Fe^3+^, Na^+^, Mg^2+^, etc., and the low proportion and concentration of Au^3+^ significantly increase the demands for the high selectivity of the adsorbents. Around 10–20% of the PCB is made of copper, which forms the conducting layer for electrical connection between different components, and which is two to three orders of magnitude higher than Au^3+^ ions [16]. We analyzed the adsorption behavior of rGO membranes in the dual-ion solution of the concentration ratio Au^3+^:Cu^2+^ = 1:1 (100 ppm). As shown in Figure 2a, the maximum adsorption capacity of Au^3+^ ions in the dual-ion solution by rGO membranes reached 0.48 g/g after 48 h, which is 87% of the maximum adsorption capacity in single Au^3+^ ion solution (0.55 g/g). The adsorption capacity of the rGO membrane to Cu^2+^ ions also reached the maximum 0.04 g/g at 48 h. It is obvious that the maximum adsorption amount of Au^3+^ ions can reach up to about 16 times that of Cu^2+^ ions in the same ion concentration. Similar results were found in the adsorption behavior of Au^3+^ in the dual-ion solution with an equal ratio of Au^3+^:Cu^2+^ (0.5 ppm). As shown in Figure 2b, the adsorption capacity of the rGO membrane for Cu^2+^ in solution was extremely low, only 0.07 mg/g at 48 h, while the adsorption capacity for Au^3+^ was 2.23 mg/g, which is 31 times that of Cu^2+^. To study the influences of different valence-state ions to the selective adsorption of an rGO membrane to Au^3+^, we performed the same selective adsorption to Au^3+^ in the dual-ion solution (100 ppm/0.5 ppm), but changed the Cu^2+^ to Fe^3+^. The results show that the maximum adsorption capacity of Au^3+^ ion (0.53 g/g) is about 10 times of that of Fe^3+^ ion (0.05 g/g) at 100 ppm (Figure 2c), and about 3 times of that of Fe^3+^ ion at 0.5 ppm (Figure 2d). Therefore, the selective adsorption of rGO membranes to Au^3+^ is extremely outstanding even if all of them are trivalent cations. In addition, we also studied the selective adsorption of the rGO membrane to Au^3+^ in the mixed solution with other ions (Co^2+^, Mg^2+^ and Na^+^) and found that rGO membranes also have excellent selectivity for Au^3+^ in mixed solutions (Figure S3).

Furthermore, we studied the selective adsorption of rGO membrane to Au^3+^ in a multi-ion solution environment. Usually, PCB leachate contains large amounts of common metals such as Cu^2+^, Na^+^, Fe^3+^ and Mg^2+^ in an acid digest [21]. Here, we prepared the mixed solution with the initial mass ratios (denoted R_i_) for Au^3+^:Cu^2+^:Na^+^:Fe^3+^:Mg^2+^ as R_i_ = 1:1000:1000:100:100, where the concentration of Au^3+^ was 100 ppm (Figure 2e). Then, the dried rGO membranes were immersed in the mixed-ion solution. As shown in Figure 2f, the adsorption capacity of rGO membranes for Au^3+^, Cu^2+^, Na^+^, Fe^3+^ and Mg^2+^ in mixed-ion solution were 261.6 mg/g, 29.4 mg/g, 17.5 mg/g, 59.1 mg/g and 18.4 mg/g, respectively. The corresponding mass ratio in the rGO membranes (denoted R_f_) of Au^3+^:Cu^2+^:Na^+^:Fe^3+^:Mg^2+^ were R_f_ = 1:0.11:0.06:0.22:0.07. The mass ratios of Au^3+^:Cu^2+^:Na^+^:Fe^3+^:Mg^2+^ in rGO membranes were four orders of magnitude higher than the initial mass ratios in the mixed-ion solution. This result further illustrates that the selective adsorption of the rGO membrane to Au^3+^ in the mixed-ion solution has excellence. The PCB permeate contains a large amount of metal ions, and the type and concentration of metal ions in different types of PCB permeates may vary. The simulated solution prepared in the laboratory may not completely mimic the actual environment, and there may be certain metal ions that selectively adsorb onto the rGO membrane, which requires further discussion and investigation.

Adsorption separation technology is one of the most efficient and economical methods for extraction, concentration and purification. High-valent metal ions, especially Au^3+^, Co^2+^, Cu^2+^, Cd^2+^, Cr^2+^ and Pb^2+^, strongly interact with graphene sheets [22] and exhibit ineffective or slow desorption [23] using conventional methods. From the actual experimental results, we found that the formed Au after adsorption is difficult to remove from the membrane. As Li et al. have demonstrated, burning the rGO membrane in air at 700 °C leaves behind gold particles, which is currently the most cost-effective method [11]. This limits the reuse of rGO membranes and needs further exploration and research in future work.

### 2.3. Adsorption Energy of Au^3+^ Ions on rGO Membrane by DFT Calculation

**Figure 3:**
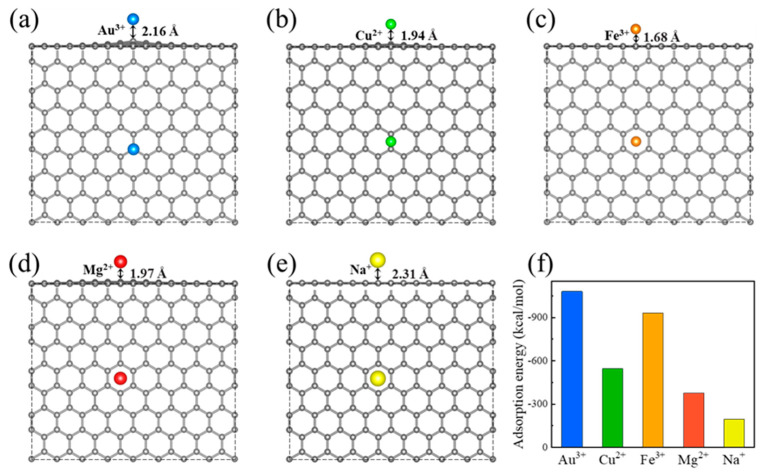
Models of ion adsorption on graphene by DFT calculations (**a**–**e**). The optimized configuration of the adsorption of Au^3+^, Cu^2+^, Fe^3+^, Mg^2+^ and Na^+^ on rGO surfaces. Gray represents the C atom, while yellow, green, brown, pink and blue represent Au, Cu, Fe, Mg and Na ions, respectively. (**f**) Adsorption energies of various cations on the graphite surface. The absolute value of the adsorption energy of Au^3+^ is the largest, which is −1080.04 kcal/mol. The absolute value of the adsorption energies of the other ions from large to small are listed as Fe^3+^ > Cu^2+^ > Mg^2+^ > Na^+^.

Li et al. attributed the excellent Au^3+^ selectivity of rGO to the graphene areas spontaneously reduce gold ions to metallic gold, and the oxidized regions allow good dispersibility of the rGO material so that efficient adsorption and reduction of gold ions at the graphene areas can be realized [11]. To explore the underlying physics, we first performed the density functional theory (DFT) calculations for a detailed study of the adsorption behavior of Au^3+^ and other ions on the rGO membrane. We constructed a model including 128 carbon atoms in a honeycomb lattice to simulate the rGO membrane. The vacuum layers were set to at least 23.4 Å to avoid interactions between periodic images. To model the adsorption between different metal cations and rGO membranes, the adsorption sites, including top, bridge and center sites, were considered as initial configurations and then the geometry optimizations were performed [24]. The optimized configuration of Au^3+^, Fe^3+^, Cu^2+^, Mg^2+^ and Na^+^ on rGO membranes is shown in Figure 3a–e.

We also calculated the adsorption energies between metal cations and graphene sheets, respectively, according to the DFT calculation results, and compared the interaction between different metal cations in mixed-ion solution and the rGO membrane. The adsorption energy is calculated using the formula:

(1) $$E_{adsorption}=E_{total}-E_{rGO}-E_{cation},$$

where $E_{total}$ is the total energy of the system, $E_{rGO}\;$ is the energy of rGO and $E_{cation}$ is the energy of the specified cation including Au^3+^, Cu^2+^, Fe^3+^, Mg^2+^ and Na^+^. As shown in Figure 3f, the absolute value of the adsorption energy of Au^3+^ is the largest, which is −1080.04 kcal/mol. The absolute value of the adsorption energies of the other ions from large to small are listed as Fe^3+^ > Cu^2+^ > Mg^2+^ > Na^+^. This result is consistent with the experimental result, which indicates that the interaction between Au^3+^ and the rGO membrane is significantly stronger than that of the other ions with the rGO membrane. In the mixed solution, due to the difference in adsorption energy, even when the proportion of ion concentration in the solution is vastly different, Au^3+^ ions are still preferentially captured by rGO membrane. Once the gold ions are adsorbed on the rGO membrane, they will spontaneously reduce to elemental form, which is consistent with the observation in our experiment. After the Au^3+^ ions on the membrane are reduced, the gold ion concentration on the rGO membrane becomes extremely low, and the concentration difference of Au^3+^ ions inside and outside the membrane is still maintained. This allows the rGO membrane to overcome the rapid equilibrium under low concentration conditions. Under the combined action of adsorption energy and oxidation reduction, the excellent selective adsorption ability of the rGO membrane is formed. The rGO membrane forms a π electron-rich structure in the form of hexagonal carbon rings, and we speculate that the cation–π interaction has an effect on the difference of adsorption energy [22,24,25]. Therefore, the strong cation–π interaction between Au^3+^ and π electrons on the membrane leads to high adsorption efficiency. And also, the difference in the adsorption energy caused by the cation–π interaction with the absolute value of Au^3+^ being the largest enables the rGO membranes to selectively adsorb Au^3+^ in the mixed solution. This inference is also consistent with the previously reported graphene material with good adsorption capacity for heavy cations, indicating that low-cost carbon-based materials with π-electron-enriched structures have broad applications in secondary metal recovery potential [22].

### 2.4. Characterization

To observe the crystal structure and chemical state of Au^3+^ ions on the rGO membranes after adsorption, we used XRD, XPS and SEM to characterize the system.

**Figure 4:**
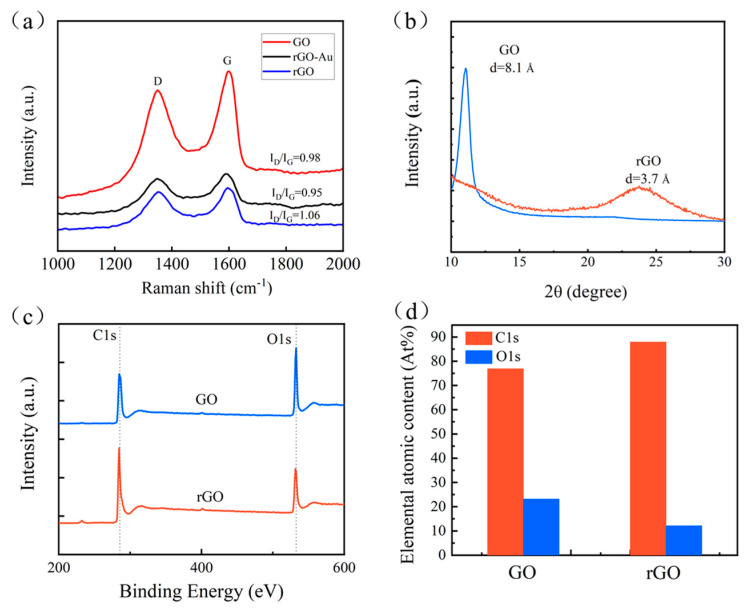
Characterization experiment for rGO membranes. (**a**) Raman spectra of the GO, rGO and rGO-Au. The ID/IG ratios of GO, rGO and rGO-Au are 0.98, 1.06 and 0.95, respectively. (**b**) XRD patterns of GO and rGO membranes. There are peaks at 10.9° and 24.1° for the GO membranes and rGO membranes, respectively. (**c**) Full−scan X−ray photoelectron spectrometer spectra of GO and rGO membranes. The C1s and O1s peak positions of membranes were at 285.08 eV and 532.08 eV, respectively. (**d**) Elemental atomic content of C and O. It is obvious that the oxygen content in each sample decreased sharply, indicating the successful removal of oxygen-containing groups from the GO surface.

**Figure 5:**
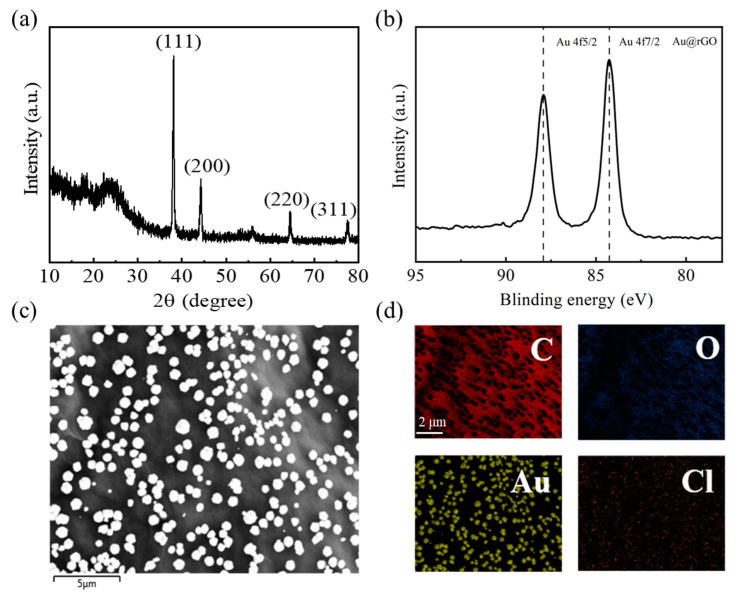
Characterization experiments of the rGO membrane after Au adsorption. (**a**) XRD patterns. The peak at ~24° is from the rGO membranes, and the peaks at ~38.1°, 44.4°, 64.5° and 77.5° are close to the value of 2θ for the (111), (200), (220) and (311) surface of gold, respectively. (**b**) XPS spectra of Au (4f), which means that the valence state of gold is 0. (**c**) SEM image shows that a large number of submicron particles with a diameter of about 1 μm appeared. (**d**) Elemental mapping of the rGO membrane after Au adsorption based on SEM analysis. The elements of C, O, Au and Cl are displayed by the colors of red, blue, yellow and green, respectively.

As shown in Figure 4, we conducted a detailed characterization of the rGO membrane used in this study and provided some of its characteristics. The Raman spectrum in Figure 4a indicates that the ID/IG ratios of GO, rGO and rGO-Au are 0.98, 1.06 and 0.95, respectively. The defect density characteristic of carbon materials, especially graphene materials, is represented by the LD, which refers to the distance between two adjacent defects. Therefore, the decrease in ID/IG observed after the extraction of gold suggests a higher defect state in rGO, supporting the electron donation from rGO to gold. We used XRD to analyze GO membranes and rGO membranes. The interlayer spacings (denoted as d) of membranes were indicated by the Bragg peaks in the XRD spectrum. There are peaks at 10.9° and 24.1° for the GO membranes and rGO membranes, respectively, as shown in Figure 5b. With the thermal reduction process, the interlayer spacing of membranes decreased from 8.0 Å to 3.7 Å. Next, XPS was utilized to analyze the oxygen group content. The XPS full-scan spectra and C1s and O1s partial spectra of GO membranes and rGO membranes are shown in Figure 4c. The C1s and O1s peak positions of membranes were at 285.08 eV and 532.08 eV, respectively. From the elemental contents of C and O in the full-scan spectra, it is obvious that the oxygen content in each sample decreased sharply (from 25.5% to 11.4%), indicating the successful removal of oxygen-containing groups from the GO surface (Figure 4d).

As shown in Figure 5a, several Bragg peaks appeared at various diffraction angles (2θ). The peak at ~24° is from the rGO membranes [26], and the peaks at ~38.1°, 44.4°, 64.5° and 77.5° are close to the value of 2θ for the (111), (200), (220) and (311) surface of gold, respectively. To further verify the chemical state of Au^3+^ ions on the membranes, we performed an XPS experiment. As shown in Figure 5b, there appear peaks at 84.24 (4f_7/2_) and 87.90 (4f_5/2_) in the XPS spectrum, which means that the valence state of gold is 0, indicating the existence of gold simple substance in the membranes.

**Figure 6:**
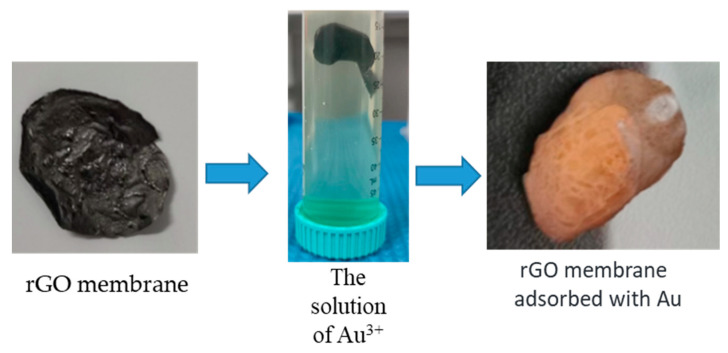
Adsorption capacity of Au^3+^ ions using rGO membranes. A schematic of separated Au^3+^ ions by rGO membranes.

In order to further analyze the structural details, Figure 5c shows the SEM image of the rGO membrane after adsorption of Au. A large number of submicron particles with a diameter of about 1 μm appeared. Then we analyzed the elemental distributions in this region, and the elemental mappings of C, O, Au and Cl in the aggregation are shown in Figure 6d. Since the elemental mapping of Au completely correlates to the distribution, shape and size of the submicron particles appearing in Figure 5c, it can be confirmed that the submicron particles with a diameter of 1 μm in Figure 5c were aggregated after the reduction of Au^3+^ adsorbed on the membranes.

## 3. Materials and Methods

### 3.1. Materials

All chemical supplies used in our work, including NaCl (99%), KCl (99%), MgCl_2_·6H_2_O (99%), CaCl_2_·2H_2_O (99%), CuCl_2_·2H_2_O (99%), FeCl_3_⋅6H_2_O (99%) and AuCl_3_ (99%), were purchased as analytical reagents from Shanghai Aladdin Biochemical Technology Co., Ltd., Shanghai, China.

### 3.2. Synthesis of Reduced Graphene Oxides Membranes

Freestanding GO membranes can be prepared by drop-casting the GO suspension (5 mg/mL, 1 mL) droplets onto a smooth paper substrate. In order to speed up the preparation process without affecting the quality of GO membranes, the freestanding GO membranes in our study were dried thoroughly at 70 °C for 12 h. After that, they were peeled off, rinsed and soaked with DI water for more than half an hour to remove the absorbed metal ions, then dried in a dry dish at room temperature for three days. These prepared freestanding membranes were used for ion-controlling and salt-solution-adsorption experiments.

These GO membranes underwent reduction by heat treatment of 180 °C for an hour to obtain the rGO membranes. The prepared GO and rGO membranes were stored in dry and clean containers before usage.

### 3.3. Gold Adsorption Measurement

As shown in Figure 6, we prepared circular rGO membranes with a diameter of approximately 15 mm and a thickness of about 0.1 mm. The specific surface area is 1.1597 m²/g. The membranes were immersed in 50 mL solutions of Au^3+^ ions with different concentrations at room temperature. The pH of the solution was 4 and the concentrations of Au^3+^ were 50, 100, 200, 500, 1000 and 2000 ppm. Throughout the whole adsorption process, the membranes were always suspended in the solution to ensure sufficient contact between the rGO membrane and the solution. After 48 h, the membranes were collected from the solution, and the solution on the surface of the membranes was removed by centrifugation. Then the membranes were dried and stored. Then the filtrates were analyzed by inductively coupled plasma optical emission spectroscopy (ICP-OES) to determine the adsorption capacity, $Q_{e}$ (mg/g). It was calculated as $Q_{e}=\frac{\left(C_{0}-C_{e}\right)\;\times \;V}{m}$ where $C_{0}$ is the initial concentration of Au (ppm), $C_{e}$ is its final concentration in the filtrate (ppm), V is the volume of the used suspension (L) and m is the mass of dry rGO membranes (g). The amount of Au^3+^ adsorbed in the rGO membrane was measured by comparing the concentration difference of the solution before and after the adsorption process (The original result is in Table S1). To ensure reproducibility, all the measurements were repeated at least 3 times.

### 3.4. Preparation of Mixed Solutions Simulating PCB Leachate

In order to simulate the real PCB leachate, we prepared the mixed-ion solution used in the experiment according to the ion ratio of Cu^2+^:Na^+^:Fe^3+^:Mg^2+^:Au^3+^ as 1000:1000:100:100:1 (100 ppm Au^3+^). An amount of 9 mg of rGO membrane was added to the solution, and the mixture was still standing for 48 h at room temperature.

### 3.5. Characterization

The XPS results of each rGO membrane after adsorption of Au were obtained using a Thermo Fisher Scientific ESCALAB 250Xi spectrometer. The metal ion content in the mixed solution was quantized using a PerkinElmer Optima 7000DV ICP-OES system. Using 40 kV and 40 mA Cu *K*α radiation, a Bruker D8 Advance XRD was used to characterize the structure of the rGO membranes. On a LEO 1530VP, the rGO membranes were mounted on carbon tape and analyzed using 10 kV with a working distance of 8.3 mm.

### 3.6. Computational Details Using Density Functional Theory

We performed structural relaxation and the electronic structure calculations based on density functional theory (DFT) calculations, as implemented in the Vienna Ab initio Simulation Package (VASP) [27]. The ion–electron interaction was treated by projector augmented-wave (PAW) method [28,29]. The exchange–correlation interaction between electrons was treated by the generalized gradient approximation (GGA) of the Perdew–Burke–Ernzerhof (PBE) [30]. An energy cutoff of a plane wave was set to 650 eV to expand the wavefunction of valence electrons (5d^10^6s^1^ for Au, 3d^10^4s^1^ for Cu, 3d^64^s^2^ for Fe, 2p^63^s^2^ for Mg, 2p^63^s^1^ for Na and 2s^22^p^2^ for C). The structural relaxations were performed until the Hellmann–Feynman forces within total energy and force convergences were 10^−7^ eV and 10^−2^ eV/Å, respectively. Gamma-centered Monkhorst–Pack grids [31] of 1 × 1 × 1 were chosen for ionic adsorption models. The Tkatchenko–Scheffler method was adopted to describe the van der Waals interactions between ions and the graphene slab. We considered three ionic adsorption sites, comprising top, center and bridge, to study the preference of different ionic adsorption rates.

## 4. Conclusions

Au recycling from secondary metal resources not only has economic value but also has a good environmental effect. In this work, inspired by the work of Li et al., we studied the selective adsorption of rGO membranes to Au^3+^ in mixed-ion solutions. Isothermal adsorption experiments showed that the adsorption capacity of Au^3+^ in the rGO membranes can reach up to 1.79 g/g. In order to further understand the excellent selectivity of Au^3+^ by rGO membranes, we conducted adsorption experiments in the dual-ion solutions of Au^3+^/Cu^2+^ and Au^3+^/Fe^3+^ of two concentrations (100 ppm and 0.5 ppm). We found that the maximum adsorption capacity of Au^3+^ was 0.48 g/g, 16 times that of Cu^2+^, in a dual-ion solution of Au^3+^/Cu^2+^ at a concentration of 100 ppm. Under the same conditions, when another ion is replaced by Fe^3+^, the maximum adsorption capacity of Au^3+^ is also up to 10 times that of Fe^3+^. By configurating the mixed-ion solution with an initial mass ratio of Au^3+^:Cu^2+^:Na^+^:Fe^3+^:Mg^2+^ = 1:1000:1000:100:100, we found that the mass ratio of Au^3+^ to other metal ions (Cu^2+^, Na^+^, Fe^3+^ and Mg^2+^) in the rGO membranes can increase by four orders of magnitude. After removing the membrane, further processing was conducted, and through XPS and SEM analysis, it was observed that the membrane was enriched with gold, and there were almost no other elements present in the membrane. To explore the underlying physics, we performed DFT calculations to understanding its selectivity. It was found that the absolute value of adsorption energy of Au^3+^ on the membrane was the highest, indicating that the interaction between Au^3+^ and the rGO membrane was obviously stronger than that of other ions with the membrane. In general, our work provides a detailed exploration about the selectivity of rGO membranes to Au^3+^ in different solution environments, and the results are beneficial for rGO membranes to be used as adsorbents to capture Au from secondary metal resources.

## Figures and Tables

**Table 1:** Adsorption capacities of various gold adsorbents.

Adsorbents	Adsorption Capacities (mg/g)	Reference
Activated carbon	40	[17]
Ion exchange resin	50	[18]
Nanofibrous membrane	250	[19]
BUT-33-PpPD	1600	[12]
COFs	1725	[14]
Reduced graphene oxide (rGO)	1850	[11]
Porphyrin-based nanoporous COP (COP-180)	1620	[20]
rGO membrane	1797	Current work

## Data Availability

The data presented in this study are available on request from the corresponding author.

## Supplementary Materials

The supporting information can be downloaded at: https://www.mdpi.com/article/10.3390/ijms241512239/s1.
