# Supplementary material for: Thermally Reduced Graphene Oxide Membranes Revealed Selective Adsorption of Gold Ions from Mixed Ionic Solutions
Source: Int J Mol Sci. 2023 Jul 31;24(15):12239. doi: 10.3390/ijms241512239 (PMC10418702; doi:10.3390/ijms241512239)
Supplement: Supplementary file 1 [file ijms-24-12239-s001.zip › ijms-2432848-supplementary.pdf]

Supplementary Information

Excellent selective adsorption of elemental gold by thermally reduced graphene oxide membranes in mixed ionic solutions

Yu Qiang[1†], Siyan Gao[1†], Yueyu Zhang[2,3], Shuai Wang[1], Liang Chen[4], Liuhua Mu[2,3], Haiping Fang[1], Jie Jiang[4]*, and Xiaoling Lei [1*]

1 School of Physics and School of Material Science and Engineering, East China University of Science and Technology, Shanghai 200237, China

2 Wenzhou Institute, University of Chinese Academy of Sciences, Wenzhou, Zhejiang 325001, China.

3 University of Chinese Academy of Sciences, Beijing 100049, China

4 School of Physical Science and Technology, Ningbo University, Ningbo 315211, China

⋆ Corresponding author. E-mail: leixiaoling@ecust.edu.cn, jiangjie1@nbu.edu.cn

Context:

PS1: Adsorption time of rGO membranes at different concentrations

PS2: Adsorption efficiency of rGO membranes at 50ml of different solution concentrations from 0.50 ppm to 300 ppm

PS3: The selective adsorption capacity of rGO membranes in the mixed solution of Au^{3+} and other ions(Co^{2+}，Mg^{2+}，Na^{+})

PS4: Adsorption capacity of rGO membranes and GO membranes

PS5: Stability experiments of rGO membranes

PS6：The results of ICP-OES

PS1: Adsorption time of rGO membranes at different concentrations

We studied the change of the adsorption amount of Au^{3+} ions with time in different concentration solutions. As shown in Fig. S1, these rGO membranes were immersed in the Au^{3+} solutions with concentrations of 0.5 ppm, 50 ppm, 100 ppm, 200 ppm, 500 ppm, 1000 ppm, and 2000 ppm, respectively. At regular intervals, 3 ml of the immersion solutions of all concentrations were taken out for ICP-OES tests, and then the mass of Au^{3+} adsorbed in the rGO membranes was calculated. The adsorption capacity of rGO membrane increases with the increase of immersion time at the very beginning, and stays as a constant after the adsorption amount was close to the maximum value around 36 h. In low concentration solutions (0.5 ppm, 10 ppm, 50 ppm, 100 ppm), the rGO membranes adsorbed almost all the gold ions in the solution within 4 h. At low concentration, there is still a gap in the adsorption rate of the rGO membranes compared with Li et al [1]., who completed 60% adsorption within the first 10min using the graphene solution. However, in high concentration solutions (> 100ppm), the rGO membranes have completed 40% of its adsorption capacity within the first 4 hours, and with the time increases to 10 h, the rGO membranes had completed half of its own adsorption capacity, which is a generalization of the work of Li et al [1] at high concentrations.

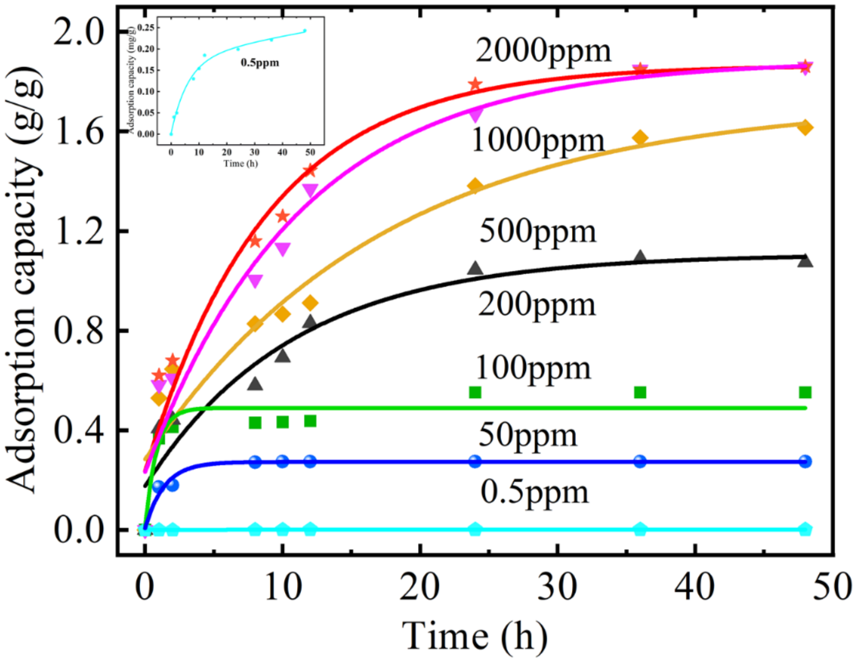

Fig. S1 Adsorption efficiency of rGO membranes for Au^{3+} with different equilibrium concentration (0.5-2000 ppm), shown in the illustration is the adsorption efficiency of 0.5ppm.

PS2: Adsorption efficiency of rGO membranes at 50ml of different solution concentrations from 0.50 ppm to 300 ppm

Fig. S2 shows the adsorption efficiency of rGO membranes at 50ml of different solution concentrations from 0.50 ppm to 300 ppm. In the solution with ion concentration of 0.5 ppm, 10 ppm, 50 ppm, 100 ppm and 200 ppm, the adsorption efficiency of rGO membranes can reach up to 97%, 96%, 94%, 96%, and 97%, respectively, which means that almost all the Au^{3+} ions in the solution were adsorbed in the rGO membranes. With the increase of the ion concentration from 200 ppm to 300 ppm, the adsorption efficiency of rGO membranes decreased gradually from 97% to 82%. Taking a commonly PCBs as an example, a typical computer circuit board, the gold content is about 250 ppm, the adsorption efficiency of rGO membranes is about 96% [2]. This means that the rGO membranes can achieve an excellent Au^{3+} adsorption efficiency when it used as an adsorbent in the PCB leachate. By limiting the concentration of Au^{3+} solution (50 ml) shows that the rGO membranes can also be used as a quality adsorbent even if the total amount of gold in the solution is not high.

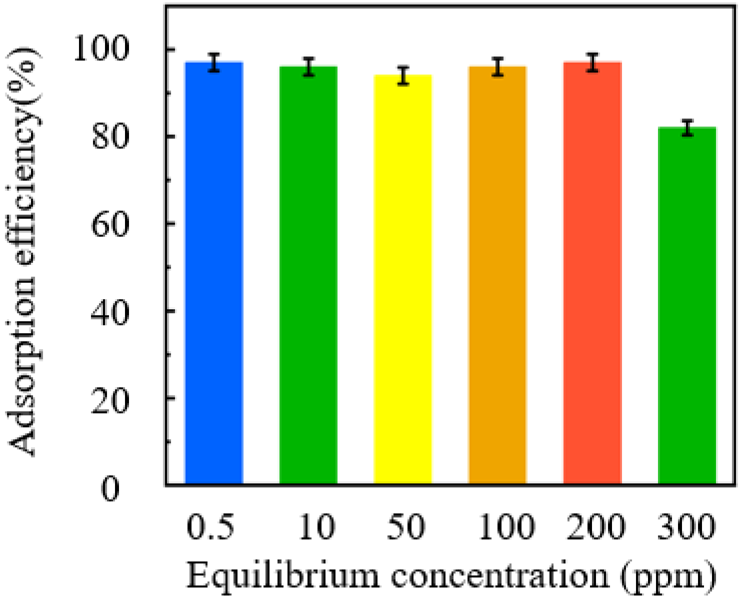

Fig. S2 Time-dependent curves of the adsorption capacity for Au^{3+} ions in different concentration solutions.

PS3: The selective adsorption capacity of rGO membranes in the mixed solution of Au^{3+} and other ions(Co^{2+}, Mg^{2+}, Na^{+})

We studied the selective adsorption capacity of rGO membranes in the mixed solution of Au^{3+} and other ions(Co^{2+}, Mg^{2+}, Na^{+}), and found that rGO membranes also have excellent selectivity for Au^{3+} in solution (Fig. S3).

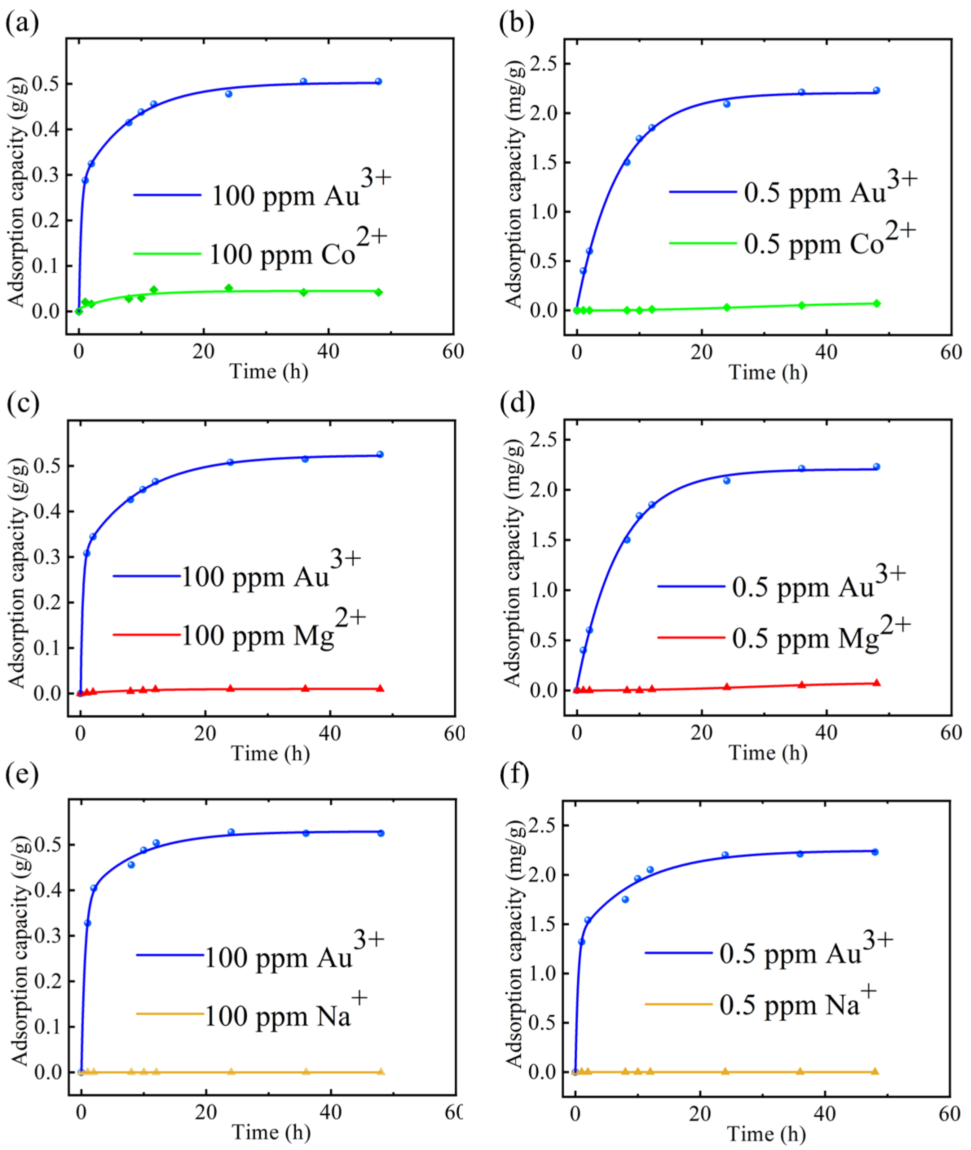

Fig. S3 (a-b), Time-dependent curves of the adsorption behavior for Au^{3+} ions in Au^{3+}/Co^{2+} mixed ion solutions. (c-d), Time-dependent curves of the adsorption behavior for Au^{3+} ions in Au^{3+}/Mg^{2+} mixed ion solutions. (e-f), Time-dependent curves of the adsorption behavior for Au^{3+} ions in Au^{3+}/Na^{+} mixed ion solutions.

PS4: Adsorption capacity of rGO membranes and GO membranes

We studied the adsorption capacity of GO and rGO respectively, with the Au^{3+} ion concentration of 100 ppm, the Au^{3+} ion adsorption capacity of GO film was 0.04 g/g, while the Au ion adsorption capacity of rGO film was 0.50 g/g (Fig. S4). The adsorption capacity of rGO membrane is about 10 times higher than that of GO. Thus, we chosen rGO membranes as adsorbent to adsorb Au^{3+} ions.

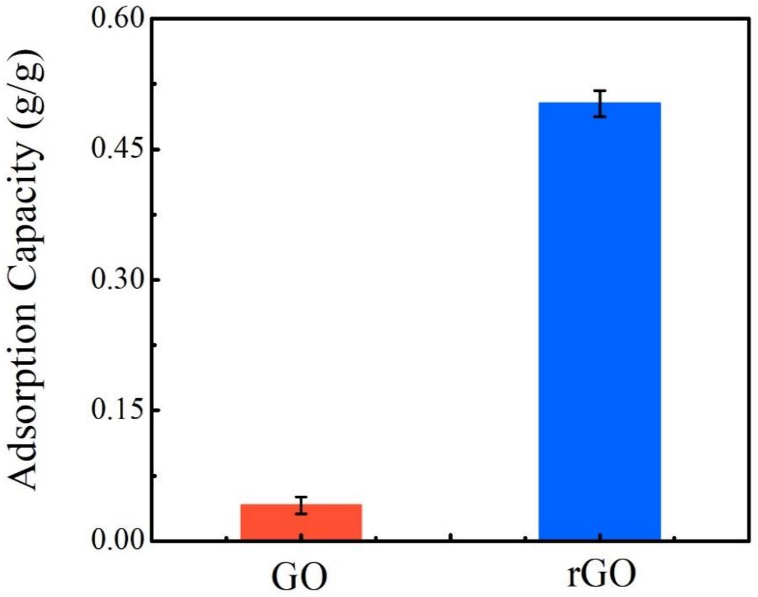

Fig S4. Adsorption capacity of rGO membranes and GO membranes

PS5: Stability experiments of rGO membranes

It is well known that GO membranes are easy to swell when immersed in aqueous solution, and this feature hinder the potential ion filtration applications of GO membranes. The rGO membranes were soaked in water for 1 day, 2 days, 5 days, 10 days, 15 days and 30 days. After stirring with a glass stirring rod, the morphology of the rGO membranes in water were recorded by photograph. As shown in Fig S5, the rGO membranes were found to be very stable after prolonged soaking. This indicates that the swelling of GO membranes in water can be well resolved to a certain extent by thermal reduction of GO membranes.

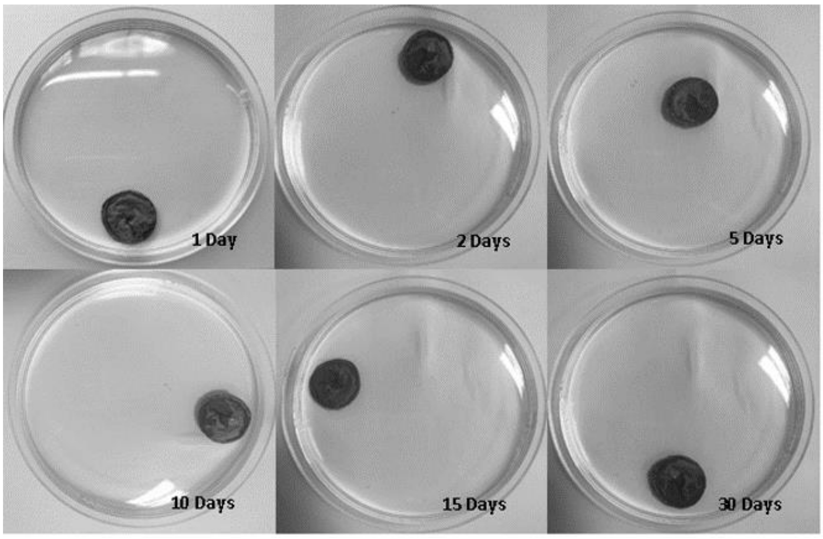

Fig. S5 Drastically different stability of GO and rGO membranes in water. (1): 1 day, (2): 2 days, (3): 5 days, (4): 10 days (5)15 days and (6) 30 days. The photos were taken after the solutions had been stirred with a glass stirring rod

PS6：The results of ICP-OES

The filtrates were analyzed by inductively coupled plasma optical emission spectroscopy (ICP-OES) to determine the adsorption capacity, Q_e (mg/g). It was calculated as $Q_e = \frac{(C_0 - C_e) \times V}{m}$/mwhere C_0 is the initial concentration of Au (ppm), C_e is its final concentration in the filtrate (ppm), V is the volume of the used suspension (L) and m is the mass of dry rGO membranes (g). The results of ICP-OES in a tabular format in Table SI

Table S1. The results of ICP-OES

Sample Name	volume (ml)	Mass （mg）	Au：C_0 - Ce （ppm）	Cu：Ce （ppm）
Au：Cu=1:1 （100ppm）0h	50	7.7	0	0
Au：Cu=1:1 （100ppm）1h	50	7.7	30.8000	0.2494
Au：Cu=1:1 （100ppm）2h	50	7.7	39.0328	0.4620
Au：Cu=1:1 （100ppm）8h	50	7.7	56.7058	0.7700
Au：Cu=1:1 （100ppm）10h	50	7.7	58.5307	1.0780
Au：Cu=1:1 （100ppm）12h	50	7.7	59.1406	1.3860
Au：Cu=1:1 （100ppm）24h	50	7.7	72.6926	1.5400
Au：Cu=1:1 （100ppm）36h	50	7.7	72.6926	1.5400
Au：Cu=1:1 （100ppm）48h	50	7.7	72.6926	1.5400
Sample Name	volume (ml)	Mass (mg)	Au：C_0 - Ce (ppm)	Cu：Ce (ppm)
Au：Cu=1:1 （0.5ppm）0h	50	8.7	0	0
Au：Cu=1:1 （0.5ppm）1h	50	8.7	0.0616	0
Au：Cu=1:1 （0.5ppm）2h	50	8.7	0.0924	0
Au：Cu=1:1 （0.5ppm）8h	50	8.7	0.2002	0
Au：Cu=1:1 （0.5ppm）10h	50	8.7	0.2772	0

Au：Cu=1:1（0.5ppm）12h	50	8.7	0.3234	0.0052
Au：Cu=1:1（0.5ppm）24h	50	8.7	0.3460	0.0104
Au：Cu=1:1（0.5ppm）36h	50	8.7	0.3672	0.0121
Au：Cu=1:1（0.5ppm）48h	50	8.7	0.3880	0.0121
**Sample Name**	**volume (ml)**	**Mass (mg)**	**Au：C_0 - Ce (ppm)**	**Fe：Ce (ppm)**
Au：Fe=1:1（100ppm）0h	50	8.3	0	0
Au：Fe=1:1（100ppm）1h	50	8.3	39.4831	3.4544
Au：Fe=1:1（100ppm）2h	50	8.3	57.1720	4.3375
Au：Fe=1:1（100ppm）8h	50	8.3	67.1586	4.6310
Au：Fe=1:1（100ppm）10h	50	8.3	72.6831	4.9086
Au：Fe=1:1（100ppm）12h	50	8.3	77.2530	6.2299
Au：Fe=1:1（100ppm）24h	50	8.3	80.9233	6.8906
Au：Fe=1:1（100ppm）36h	50	8.3	88.7884	8.6120
Au：Fe=1:1（100ppm）48h	50	8.3	88.7884	8.612
**Sample Name**	**volume (ml)**	**Mass (mg)**	**Au：C_0 - Ce (ppm)**	**Fe：Ce (ppm)**
Au：Fe=1:1（0.5ppm）0h	50	9.2	0	0
Au：Fe=1:1（0.5ppm）1h	50	9.2	0.0736	0
Au：Fe=1:1（0.5ppm）2h	50	9.2	0.0920	0
Au：Fe=1:1（0.5ppm）8h	50	9.2	0.2392	0.0184
Au：Fe=1:1（0.5ppm）10h	50	9.2	0.2833	0.0368
Au：Fe=1:1（0.5ppm）12h	50	9.2	0.3404	0.0552
Au：Fe=1:1（0.5ppm）24h	50	9.2	0.3661	0.1104

Au：Fe=1:1（0.5ppm）36h	50	9.2	0.4066	0.1472
Au：Fe=1:1（0.5ppm）48h	50	9.2	0.4471	0.1840

**Sample Name**	**volume（ml）**	**Mass（mg）**	**Au：C_0 - Ce（ppm）**	**Cu：Ce（ppm）**	**Fe：Ce（ppm）**	**Na：Ce（ppm）**	**Mg：Ce（ppm）**
mixed ion solution 0h	50	9.1	0	0	0	0	0
mixed ion solution 48h	50	9.1	47.6112	5.3508	10.7562	3.185	3.3488

Supplementary References

1. Fei Li, Jiuyi Zhu, Pengzhan Sun, Mingrui Zhang, Zhenqing Li, Dingxin Xu,Xinyu Gong, Xiaolong Zou, A. K. Geim , Yang Su, and Hui-Ming Cheng. Nature communications. 2022,13,4472
2. Hagelüken, C. Acta Metall. Slovaca 2006,12, 111
